# Independent research and development, technology accumulation and innovation performance: Evidence from China’s pharmaceutical manufacturing industry

**DOI:** 10.1371/journal.pone.0266768

**Published:** 2022-04-07

**Authors:** Su Wang, Qiang Liu, Yuwen Chen

**Affiliations:** School of Business Administration, Shenyang Pharmaceutical University, Shenyang, China; University of Almería, SPAIN

## Abstract

Technology accumulation plays a vital role in research and development (R&D) activities. This paper used technology accumulation as the threshold variable to construct a dynamic panel threshold model to explore the technology accumulation threshold effect of independent R&D on innovation performance of China’s pharmaceutical manufacturing industry over the period 1995~2019. The results show that the independent R&D of China’s pharmaceutical manufacturing industry had a significant promotion effect on innovation performance. However, it is constrained by the level of technology accumulation, showing a significant double threshold effect of technology accumulation. A moderate level of technology accumulation was more conducive to independent R&D to drive innovation performance, while lower or higher technology accumulation will weaken independent R&D promotion on innovation performance. This study provided insights for policymakers and managers in making effective innovation strategies.

## Introduction

In the era of innovation-driven development of the knowledge economy, having core technologies and independent intellectual property rights play a vital role in transforming the economic development mode and improving industrial competitiveness [1]. The “New Economic Growth Theory” believes that technological progress is the source of economic growth, and independent R&D (research and development) and technology introduction are two important ways of technological progress [2]. With the continuous development of economic globalization, technology introduction and imitation have high barriers and monopolies; long-term reliance on technology introduction and imitation to promote development not only reduces the ability of independent innovation but also falls into "laggard-introduce- laggard again-introduce again" [1]. The lousy cycle state is not conducive to the sustainable development of the country and enterprises [3]. Independent R&D can not only realize original innovation and knowledge creation but also further enhance the ability of countries and enterprises to introduce and absorb technology, thereby gaining competitive advantages and innovation-driven sustainable growth [4].

As a typical R&D-driven and highly capital-intensive industry, the pharmaceutical manufacturing industry is the core force for countries to achieve independent R&D and innovation-driven development [5]. In recent years, as countries continue to increase investment in R&D, the impact of independent R&D on innovation performance has attracted the attention of many scholars, and they have discovered that technology accumulation is the basis of innovation activities and is closely related to independent R&D driving innovation performance [6]. However, existing studies on the relationship between independent R&D and innovation performance have not focused on the differences in the level of technology accumulation between regions. Based on the above background, the current study aims to estimate the impact of technology accumulation on independent R&D and innovation performance of China’s pharmaceutical manufacturing industry. It is of great significance for government departments to issue relevant policies and pharmaceutical companies to make reasonable R&D decisions.

The contributions of the current study are as follows. First, independent R&D, innovation performance, and technology accumulation are brought into the same research framework. Most existing studies have only focused on the impact of independent R&D on innovation performance and neglected the role of technology accumulation. Second, most existing studies have used the static panel threshold model and neglected the dynamic and continuous nature of the innovation process. In this study, we construct a dynamic panel threshold model to study the impact of independent R&D on innovation performance under different levels of technology accumulation. Finally, due to the specificity and complexity of drug R&D innovation, the relevant research results in other fields are less useful for the pharmaceutical manufacturing industry. The current study takes the pharmaceutical manufacturing industry as the research object, and the results and suggestions obtained are more relevant and beneficial to pharmaceutical companies in making effective innovation decisions.

## Literature review

### Independent R&D and innovation performance

Some studies acclaim that independent R&D has a positive impact on innovation performance and has a lagging property. For example, Chauvin and Hirschey [7] used corporate data from the United States, Australia, and Japan to conduct empirical research on the relationship between independent R&D investment and innovation performance. The results found that the independent R&D investment of enterprises can significantly improve innovation performance. Kang and Feng [8] used a multiple regression model and a multinomial distributed lag model to study the relationship between independent R&D investment and China’s pharmaceutical manufacturing industry performance. It turns out that R&D investment has a significant role in promoting interest-bearing performance and lags.

Some studies acclaim that independent R&D does not significantly impact innovation performance or even has a negative effect. Lin, Lee and Hung [9] used 258 technology-supported companies in the United States as the research object and used a mixed linear model to conduct empirical analysis and found that the relationship between R&D investment intensity and innovation performance is not significant. Guo [10] conducted an empirical analysis with the software industry as the research object and found that the intensity of R&D investment has a significant negative effect on both the profit and output rates.

Some studies acclaim that independent R&D has a non-linear impact on innovation performance. Soete [11] used US corporate data from 1975 to 1976 as a research sample and found a significant inverted U-shaped relationship between corporate independent R&D investment and innovation performance that first increases and then decreases. Han and Hui [12] used the static panel threshold model to conduct an empirical analysis of the impact of R&D investment on the innovation performance of China’s strategic emerging enterprises. The results show that corporate R&D investment intensity impacts performance with significant non-linear effects in strategic emerging enterprises with different ownerships. According to the study of Duan, Zhang and Hu, constantly increasing R&D investment is not a wise decision, the investment beyond the critical value does not produce a proportional return on investment, and it is very likely to become a sunk cost and cannot be fully converted into output [5].

### Technology accumulation

More relevant to our paper is that some studies have researched the relationship between technology accumulation, independent R&D, and innovation performance. At present, there are few related studies in the pharmaceutical industry, but some research results in other fields can be seen. Brucker [13] believes that technological accumulation is an indispensable resource for R&D and innovation, and the spillover effect of technological accumulation can inhibit the decrease of marginal productivity. Gao, An, and Liu [14] studied the relationship between tacit knowledge accumulation and R&D innovation. They concluded that tacit knowledge accumulation is the foundation and essential driving force of enterprise R&D and innovation activities, which can most effectively improve enterprise performance. Leonard-Barton [15] believes technological accumulation is not always conducive to innovation and may produce organizational rigidity and dependence on the technological path. Lendner and Schindler [16] found that technology accumulation plays a negative role in the relationship between R&D investment and performance, as the level of technology accumulation increases, the marginal benefit of R&D investment gradually decreases. Guo and Sun [17] used the panel data of 1470 high-tech companies to conduct multiple regression analysis and test the moderating effect of technology accumulation and obtained similar conclusions.

However, some studies have reached different conclusions. Liu [18] researched listed companies in four technology-intensive industries and found that the higher the degree of technology accumulation, the influence of independent R&D investment on innovation performance gradually increases. Chen and Hou [1] found that the relationship between independent R&D innovation and technological performance in China’s high-tech industry is significantly affected by the level of regional knowledge accumulation. As the threshold for knowledge accumulation increases, the impact of independent R&D innovation on technological performance gradually improves.

## Research design

### Model description

To measure the impact of R&D and knowledge spillovers on productivity, Griliches [19] proposed the concept of a knowledge production function with the underlying assumption that the output of the innovation process is treated as a function of R&D inputs. Since the current study aims to explore the impact of independent R&D on innovation performance, which is similar to the assumption of the knowledge production function, the model is set up by drawing on this functional framework and expressed by the Cobb-Douglas production function as:

$${R\&D}_{\mathrm{output}}=\alpha {\left({R\&D}_{\mathrm{input}}\right)}^{\beta }$$ (1)

In subsequent research, Jaffe [20] believed that innovation investment should include R&D expenditure investment and personnel investment, so the knowledge production function was improved as:

$$Q_{\mathrm{it}}=A_{\mathrm{it}}{\mathrm{RD}}_{\mathrm{it}}^{\alpha }{\mathrm{RDP}}_{\mathrm{it}}^{\beta }$$ (2)

Among them, i represents the province, t represents the year. Q_it_ is the innovation performance. A_it_ is the total factor productivity, which refers to other factors that affect innovation performance except for the funding and personnel input. RD_it_ is independent R&D investment. RDP_it_ is R&D personnel investment. α and β are the output elasticity coefficients of expenditure and personnel input. Related research shows that technology introduction, technology purchase, and technological transformation are also important factors affecting the innovation performance of pharmaceutical manufacturing industry [21–23], so this paper assumes:

$$A_{\mathrm{it}}={\mathrm{TI}}_{\mathrm{it}}^{\beta_{1}}{\mathrm{TS}}_{\mathrm{it}}^{\beta_{2}}{\mathrm{TR}}_{\mathrm{it}}^{\beta_{3}}e^{u_{i}+v_{t}+\varepsilon_{\mathrm{it}}}$$ (3)

Substituting (3) into (2) and take the logarithm of both sides. Considering the dynamics and continuity of innovation activities, since the previous innovation performance can lay the foundation for future R&D activities, the first-order lag term of innovation performance is introduced to establish the following dynamic panel model:

$${\mathrm{lnQ}}_{\mathrm{it}}={\mathrm{\alpha lnRD}}_{\mathrm{it}}+{\mathrm{\beta lnRDP}}_{\mathrm{it}}+\beta_{1}{\mathrm{lnTI}}_{\mathrm{it}}+\beta_{2}{\mathrm{lnTS}}_{\mathrm{it}}+\beta_{3}{\mathrm{lnTR}}_{\mathrm{it}}+{\mathrm{\gamma lnQ}}_{\mathrm{it}-1}+u_{i}+v_{t}+\varepsilon_{\mathrm{it}}$$ (4)

In order to study whether the level of technological accumulation can affect the relationship between independent R&D and innovation performance, the following dynamic panel single-threshold model is constructed:

$${\mathrm{lnQ}}_{\mathrm{it}}=\mathrm{\delta +}{\mathrm{\theta lnQ}}_{\mathrm{it}‐1}+{\alpha_{1}\mathrm{lnRD}}_{\mathrm{it}}I({\mathrm{TA}}_{\mathrm{it}}\leq \gamma )+{\alpha_{2}\mathrm{lnRD}}_{\mathrm{it}}I({\mathrm{TA}}_{\mathrm{it}}\mathrm{>\gamma}){+\mathrm{\beta lnRDP}}_{\mathrm{it}}+\beta_{1}{\mathrm{lnTI}}_{\mathrm{it}}+\beta_{2}{\mathrm{lnTS}}_{\mathrm{it}}+\beta_{3}{\mathrm{lnTR}}_{\mathrm{it}}+u_{i}+v_{t}+\varepsilon_{\mathrm{it}}$$ (5)

Among them, δ is a constant term. Q_it-1_ is the innovation performance of province i in t-1 year. RD_it_ is independent R&D investment. I(∙) is an indicator function, when the expression in brackets is established, I(∙) = 1, otherwise I(∙) = 0. TA_it_ is technology accumulation. *γ* is the threshold. RDP_it_ is R&D personnel investment. TI_it_ is technology introduction expenditure. TS_it_ is technology purchase expenditure. TR_it_ is technological transformation expenditure. u_i_ is the individual fixed effect, v_t_ is the time fixed effect, ε_it_ is the error term. To expand the dynamic panel double threshold model:

$${\mathrm{lnQ}}_{\mathrm{it}}=\mathrm{\delta +}{\mathrm{\theta lnQ}}_{\mathrm{it}‐1}+{\alpha_{1}\mathrm{lnRD}}_{\mathrm{it}}I({\mathrm{TA}}_{\mathrm{it}}\leq \gamma_{1})+{\alpha_{2}\mathrm{lnRD}}_{\mathrm{it}}I({\gamma_{1}\mathrm{<TA}}_{\mathrm{it}}\leq \gamma_{2})+{\alpha_{3}\mathrm{lnRD}}_{\mathrm{it}}I({\mathrm{TA}}_{\mathrm{it}}>\gamma_{2}){\mathrm{+\beta lnRDP}}_{\mathrm{it}}+\beta_{1}{\mathrm{lnTI}}_{\mathrm{it}}+\beta_{2}{\mathrm{lnTS}}_{\mathrm{it}}+\beta_{3}{\mathrm{lnTR}}_{\mathrm{it}}+u_{i}+v_{t}+\varepsilon_{\mathrm{it}}$$ (6)

Among them, γ_1_ and γ_2_ are the double threshold values, and other variables are the same as formula (5).

### Estimation method

Since individual fixed effects are specific to each province in the dynamic panel threshold model set in this paper, it needs to be eliminated before estimation. Elimination of individual effects usually uses two methods: first-order difference and intra-group transformation. However, the use of the first-order difference method easily leads to a negative correlation between the error terms, so that the distribution theory proposed by Hansen cannot be directly applied to the dynamic panel model [24]. When using the within-group transformation method to eliminate, because the lag term of the explained variable in the dynamic panel threshold model is serially correlated with the average value of the individual error term, the estimated results of the within-group transformation will be inconsistent. In view of the shortcomings of the above two methods, this paper refers to the research of Kremer [25] and uses the forward orthogonal dispersion transformation method to eliminate the fixed effect. Each observation value is subtracted from the average value of all observation values. In order to avoid the problem of sequence correlation in the error term. The forward orthogonal dispersion transformation is performed on each variable in the model (6), and the transformation model of the error term is:

$$\varepsilon_{\mathrm{it}}^{*}=\sqrt{\frac{T‐t}{T‐t+1}}\left[\varepsilon_{\mathrm{it}}‐\frac{1}{T‐t}(\varepsilon_{i(t+1)}+\cdots +\varepsilon_{\mathrm{iT}})\right],\;t=1,\cdots ,\;T‐1$$ (7)

The forward orthogonal dispersion transformation form of other variables is consistent with the error term. Therefore, the transformed error term does not have sequence correlation, and the unit matrix form of its variance is as follows:

$$\mathrm{Var}(\varepsilon_{i})=\sigma^{2}I_{T}\Rightarrow \mathrm{Var}(\varepsilon_{i}^{*})=\sigma^{2}I_{T‐1}$$ (8)

Since the first-order lagging behind the explanatory variable added by the dynamic panel threshold model has strong endogeneity. This paper uses Caner and Hansen’s method of including the endogenous explanatory variable cross-sectional data threshold model to estimate the dynamic panel threshold model [26]. There are three steps:

First, estimate a simplified equation. Use panel data least square method to estimate the simplified equation of the endogenous variableQ_it-1_ and the instrumental variable x_it_: Q_it-1_ = g(x_it_,Π)+v_it_. Get the simplified coefficient estimate $\hat{\Pi }$ and predict Q_it-1_, get the fitted value ${\hat{Q}}_{\mathrm{it}‐1}$, and substitute ${\hat{Q}}_{\mathrm{it}‐1}$ as the instrumental variable of Q_it-1_ into the panel threshold Model.

Second, estimate the threshold. The variables are sorted according to the threshold variable (TA_it_), and the threshold value γ is taken one by one after sorted TA_it_ into the dynamic panel threshold model that has eliminated individual effects, and the panel least squares method is used to estimate the residual sum of squares S(λ), where TA_it_ that minimizes the residual sum of squares is the threshold estimate $\hat{\gamma }$. When the significance level is ρ, the confidence interval of the threshold can be obtained by $\varphi =\{\gamma :{\mathrm{LR}}_{n}(\gamma )\leq C(\rho )\}$, then further confirm the accuracy of the threshold estimate, ${\mathrm{LR}}_{n}=n[S_{n}(\gamma )‐S_{n}(\hat{\gamma })]/S_{n}(\hat{\gamma }),\;C(\rho )=‐2\log(1‐\sqrt{1‐a})$.

Finally, estimate the threshold coefficient. After obtaining the estimated threshold $\hat{\gamma }$, the sample can be divided into two intervals: ${\mathrm{TA}}_{\mathrm{it}}\leq \hat{\gamma }$ and ${\mathrm{TA}}_{\mathrm{it}}>\hat{\gamma }$. Based on the threshold estimated value $\hat{\gamma }$ and the instrumental variable x_it_, the panel data Generalized Moment (GMM) is used to estimate the threshold coefficients ${\hat{\alpha }}_{1}$ and ${\hat{\alpha }}_{2}$.

### Variable selection

#### Dependent variable: Innovation performance (Q)

The indicators used to measure innovation performance in existing research mainly include the number of patents and the sales revenue of new products [21]. The number of patents is only the intermediate output of innovative activities and does not cover the entire innovation process. In addition, some innovative activities have not applied for patents. The sales revenue of new products is the direct result of the enterprise’s innovation achievements participating in the market competition, and it reflects the market value of the innovation achievements relatively objectively. Therefore, this paper selects new product sales revenue as a measure of innovation performance and uses the ex-factory price index of industrial products to deflate it and convert it to the actual value based on 1995.

#### Independent variable: Independent R&D (RD)

Independent R&D is the independent innovation activities of enterprises relying on their strength. It is the primary way for the country and industry to achieve technological progress [27]. This paper uses R&D capital stock to measure the independent R&D level of each province and uses the perpetual inventory method for calculation [28]. The formula is as follows:

$${\mathrm{RD}}_{\mathrm{it}}=(1‐\delta ){\mathrm{RD}}_{\mathrm{it}‐1}+E_{\mathrm{it}}$$ (9)

Among them, RD_it_ and RD_it-1_ are the R&D capital stock in year t and t-1 respectively in region i. δ is the depreciation rate, with a value of 15% [29]. E_it_ represents region i internal expenditures of R&D funds in year t. The R&D price index (0.55×consumer price index+0.45×fixed asset investment price index) was constructed [30], and the internal expenditures of R&D funds in each province were flattened to the actual value with 1995 as the base period. It is also necessary to determine each province’s base period R&D capital stock in China’s pharmaceutical manufacturing industry. The calculation formula is:

$${\mathrm{RD}}_{i0}=E_{i0}/\left(g+\delta \right)$$ (10)

Among them, RD_i0_ represents the R&D capital stock of region i in 1995. E_i0_ represents the internal expenditure of R&D expenditure in region i in 1995. g represents the average annual growth rate of internal expenditure of R&D expenditure in region i from 1995 to 2019. δ is the depreciation rate.

#### Threshold variable: Technology accumulation (TA)

Technology accumulation refers to the gradual accumulation of technological knowledge and results obtained by enterprises in the long-term production and innovation process [31]. The patent is an essential source of technical information in innovation activities, and it is also the primary indicator reflecting the technological level of the industry. This paper selects the stock of patent applications as a measure of technology accumulation and adopts the perpetual inventory method for calculation, similar to calculating R&D capital stock.

#### Control variable

Dependent variable lags by one period (Q_it-1_): As an endogenous first-order lag variable to control the continuity and dynamic characteristics of the innovation performance of the pharmaceutical manufacturing industry.

R&D personnel investment (RDP): The full-time equivalent of R&D personnel obtained by using the combined conversion of R&D personnel working hours and full-time personnel as a measure of R&D personnel input [32].

Technology Introduction (TI): Technology introduction (TI): As the expenditures for digestion and absorption are entirely used for technology introduction [23], this paper combines the two as a measure of technology introduction [33].

Technology purchase (TS): Mainly refers to the expenditures used to purchase the scientific and technological achievements of other units in the country. The expenditures for the purchase of domestic technology expenditures are selected as the measurement index.

Technological transformation (TR): The expenditure for technological transformation is selected as the measurement index [34].

### Data sources

The original index data in this paper are from the "China High-tech Industry Statistical Yearbook" and the provincial and municipal statistical yearbooks. The original data for the factory price index of industrial products and the calculation of the R&D price index is from the "China Statistical Yearbook". The Statistical Yearbook is China’s official database, which is authoritative and complete. Based on the statistical yearbook, scholars have conducted many reliable studies on various industries in China. The 2018 version of the "China High-tech Industry Statistical Yearbook" has not been released. Therefore, the 2017 indicator data comes from the provincial statistical yearbooks, and some missing data are imputed using the average of the two years before and after the data [35]. The final sample is the inter-provincial panel data of the pharmaceutical manufacturing industry from 1995 to 2019 in 27 provinces and cities in China (the index data of Tibet, Qinghai, Ningxia, and Xinjiang are severely missing and removed). The data processing and empirical analysis in this paper are all completed using STATA16, which takes the natural logarithm of the related variables to control the heteroscedasticity of the model. The descriptive statistical results of each variable are shown in Table 1.

**Table 1:** Descriptive statistics.

Variable	N	Mean	S.D.	Min	Max
**lnQ**	675	11.676	2.205	4.084	15.967
**lnRD**	675	10.121	2.204	2.674	14.792
**lnTA**	675	5.267	2.020	0.678	9.469
**lnRDP**	675	6.745	1.524	1.099	9.830
**lnTI**	675	8.629	1.856	0.967	12.917
**lnTR**	675	10.741	1.326	6.357	13.815
**lnTS**	675	7.878	1.787	2.166	12.085

## Empirical analysis

### Stationarity test

In order to avoid the pseudo-regression phenomenon of non-stationary variables, the stationarity of the panel data should be tested before regression to ensure the validity of the estimated results. In order to enhance the reliability of the test, this paper uses both the homogeneous unit root test (LLC) and the heterogeneous unit root test (Fisher-ADF) to test the stationarity of each variable. The test results are shown in Table 2.

**Table 2:** Panel data stationarity test results.

Variable	LLC		Fisher-ADF	
	Statistics	P	Statistics	P
**lnQ**	-3.434	0.000	203.875	0.000
**lnRD**	-9.597	0.000	163.474	0.000
**lnTA**	-5.423	0.000	151.533	0.000
**lnRDP**	-4.473	0.000	189.823	0.000
**lnTI**	-14.686	0.000	217.892	0.000
**lnTR**	-4.811	0.000	177.123	0.000
**lnTS**	-3.924	0.000	219.773	0.000

From the results of unit root tests in Table 2, each variable rejects the non-stationary null hypothesis at the 1% significance level, indicating that the original series of related variables are all stationary.

### Threshold effect test and threshold value estimation

According to the above explained dynamic panel threshold estimation method. Firstly, the related variables are changed in the forward orthogonal dispersion to eliminate the individual effects. Secondly, Use Q_it-1_ to estimate the instrumental variables and exogenous variables by panel least squares method to obtain the fitted value ${\hat{Q}}_{\mathrm{it}‐1}$, and use ${\hat{Q}}_{\mathrm{it}‐1}$ as the proxy of Q_it-1_ Variables are put into the threshold model to solve the problem of endogeneity. Finally, use Hansen’s method to determine the threshold and confidence interval. This paper uses technology accumulation as the threshold variable to test the threshold effect in sequence under different threshold numbers, and the obtained F value and self-sampling P-value are shown in Table 3.

**Table 3:** Threshold effect test results.

Model	F	BS	1%	5%	10%
**Single threshold**	24.560**	500	15.586	19.458	27.623
**Double threshold**	21.970**	500	15.196	19.032	24.292
**Triple threshold**	14.010	500	26.276	30.659	42.975

Note

** p < 0.05.

In Table 3, the single threshold F value is 24.560, and the self-sampling P-value is significant at the 5% level; the double threshold F value is 21.970, and the self-sampling P-value is significant at the 5% level; the triple threshold self-sampling result is not significant. Therefore, the dynamic panel threshold model has a significant dual-threshold effect on technology accumulation. The independent research and development of China’s pharmaceutical manufacturing industry have a significant technology accumulation threshold effect on innovation performance. Further, determine the threshold estimate and confidence interval, and the results are shown in Table 4.

**Table 4:** Threshold estimates and confidence intervals.

**Model**	**Threshold estimate**	**95% confidence interval**
**Single threshold**	1.302	[1.216, 1.332]
**Double threshold**	3.092	[3.044, 3.133]

**Fig 1:**
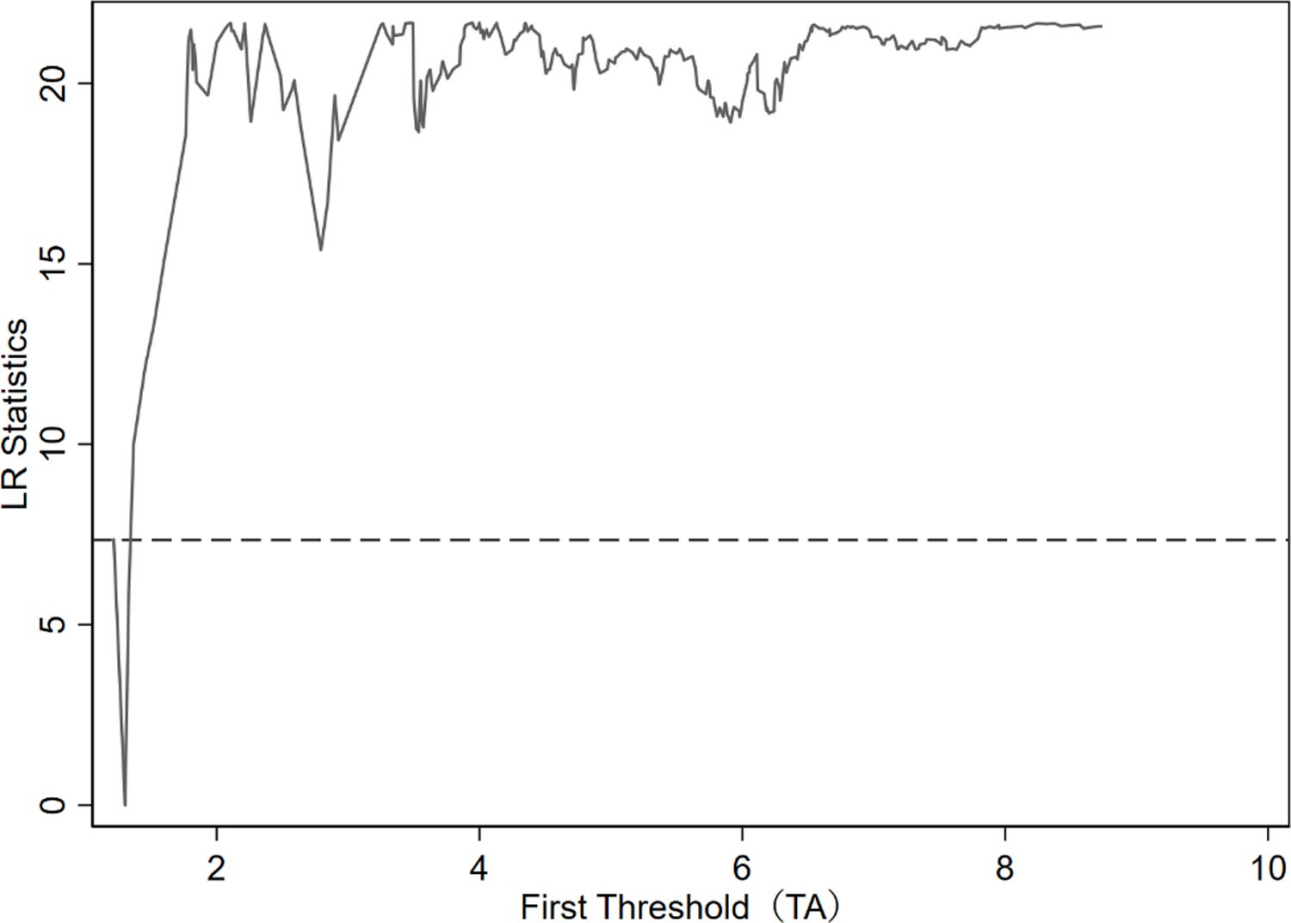
The first threshold estimate and confidence interval.

**Fig 2:**
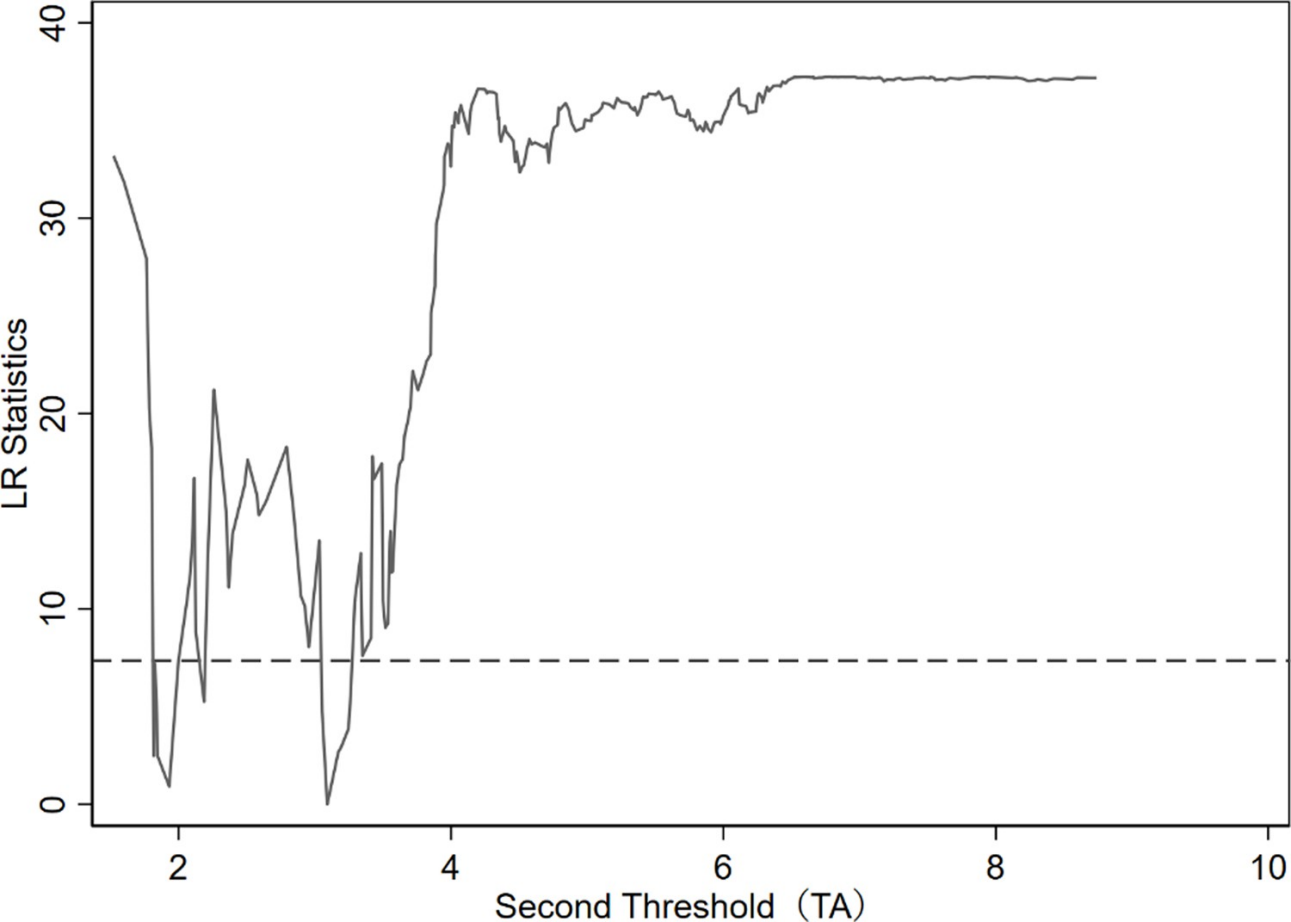
The second threshold estimate and confidence interval.

It can be seen from Table 4 that independent R&D has a significant dual-threshold effect of technology accumulation on innovation performance. The two thresholds are 1.302 and 3.092, located at 95% confidence intervals [1.216, 1.332] and [3.044, 3.133]. In order to understand the construction of the threshold estimate and the 95% confidence interval more intuitively, the likelihood ratio function graph is used to verify the authenticity of the threshold estimate, and the results are shown in Figs 1 and 2.

It can be seen from Figs 1 and 2 that when the double threshold estimates are 1.302 and 3.092, the likelihood ratio statistic LR value is 0. The 95% confidence interval of the two threshold estimates is that all LR values are less than 5%. The interval formed by the TA of the critical value (corresponding to the dotted line in the figure) at the significance level indicates that the two threshold estimates are equal to the actual value. Therefore, the sample can be divided into low-tech accumulation (TA≤1.302), medium-tech accumulation (1.302<TA≤3.092), and high-tech accumulation (TA>3.092).

### Dynamic panel threshold parameter estimation

The dynamic panel threshold model parameters are estimated based on three sample intervals divided by two threshold values. Because the model contains the explained variable lags behind the endogenous problem, if the traditional least square method and fixed effects are used to estimate the result will be biased, and the dynamic GMM estimation can overcome the endogenous problem of the model, which mainly includes the system GMM and differential GMM. Under the condition of limited samples, the system GMM has a smaller estimation deviation than the differential GMM, which can solve the problem of weak instrumental variables and is more efficient [36]. Therefore, this paper chooses the systematic GMM method to estimate the dynamic panel threshold model parameters, and the results are shown in Table 5.

**Table 5:** Dynamic panel threshold model parameter estimation results.

Variable	coefficient	S.E.	z	95% confidence interval	
**L1.Q**	0.300***	0.054	5.560	0.194	0.405
**RDP**	0.367***	0.045	8.090	0.278	0.456
**TI**	0.021	0.045	0.480	-0.066	0.109
**TS**	0.073**	0.031	2.390	0.013	0.133
**TR**	-0.190	0.334	-0.570	-0.844	0.465
**TA≤1.302**	0.235**	0.099	-2.420	-0.433	-0.045
**1.302<TA≤3.092**	0.474***	0.091	5.240	0.296	0.651
**TA>3.092**	0.315***	0.038	-4.150	-0.233	-0.084
**_cons**	-0.091**	0.046	-1.990	-0.180	-0.001

Notes

** p < 0.05

***p < 0.01.

From Table 5, it can be seen that when the regional technology accumulation level is lower than the threshold value of 1.302 (low technology accumulation interval), the influence of independent R&D on innovation performance is significantly positive at the level of 5%. As technological accumulation increases to 1.302~3.092 (medium technology accumulation interval), the positive impact of independent R&D on innovation performance increased, and it was significant at the level of 1%, indicating that increasing independent R&D investment at this level is conducive to further improving innovation performance. When technology accumulation is higher than the threshold of 3.092 (high-tech accumulation interval), the positive impact of independent R&D on innovation performance weakens and is significant at the 1% level. On the whole, the independent R&D investment in China’s pharmaceutical manufacturing industry has a significant role in promoting innovation performance, but the level of regional technology accumulation constrains this promotion. As the level of regional technology accumulation continues to increase, independent R&D has a significant impact on innovation performance. The promotion effect first strengthened and then weakened, showing a significant threshold characteristic. In a statistical sense, when the level of technology accumulation is between 1.302 and 3.092 (medium technology accumulation interval), independent R&D investment has the most significant positive impact on innovation performance.

Regarding the control variables, R&D personnel input, technology introduction, and technology purchase positively impact innovation performance, but technology introduction does not significantly promote innovation performance, while technological transformation has an insignificant negative impact on innovation performance. The results show that R&D personnel investment and technology purchase are essential factors to improve innovation performance. The improvement of China’s pharmaceutical manufacturing industry’s innovation performance mainly comes from independent R&D and domestic technology purchase, rather than technology introduction through international trade and other channels. The reason may be that the purchased domestic technology can be quickly absorbed and converted into new products to meet market demand, but the ability to absorb and absorb foreign technology is weak and cannot be effectively converted into output [34]. The explained variable is significantly positive at the 1% level for the first period. The previous period has a significant positive effect on the current innovation performance, so it is reasonable to control the dynamic impact of innovation performance and build a dynamic panel threshold model. Finally, the Sargan test results (Prob> chi2 = 1.000) show that the instrumental variables are reasonable and effective. The test results in Table 6 show that it is reasonable to use the system GMM for parameter estimation.

**Table 6:** AR(1) and AR(2) results.

Order	z	P > z
**AR (1)**	-2.201	0.028
**AR (2)**	1.010	0.313

**Fig 3:**
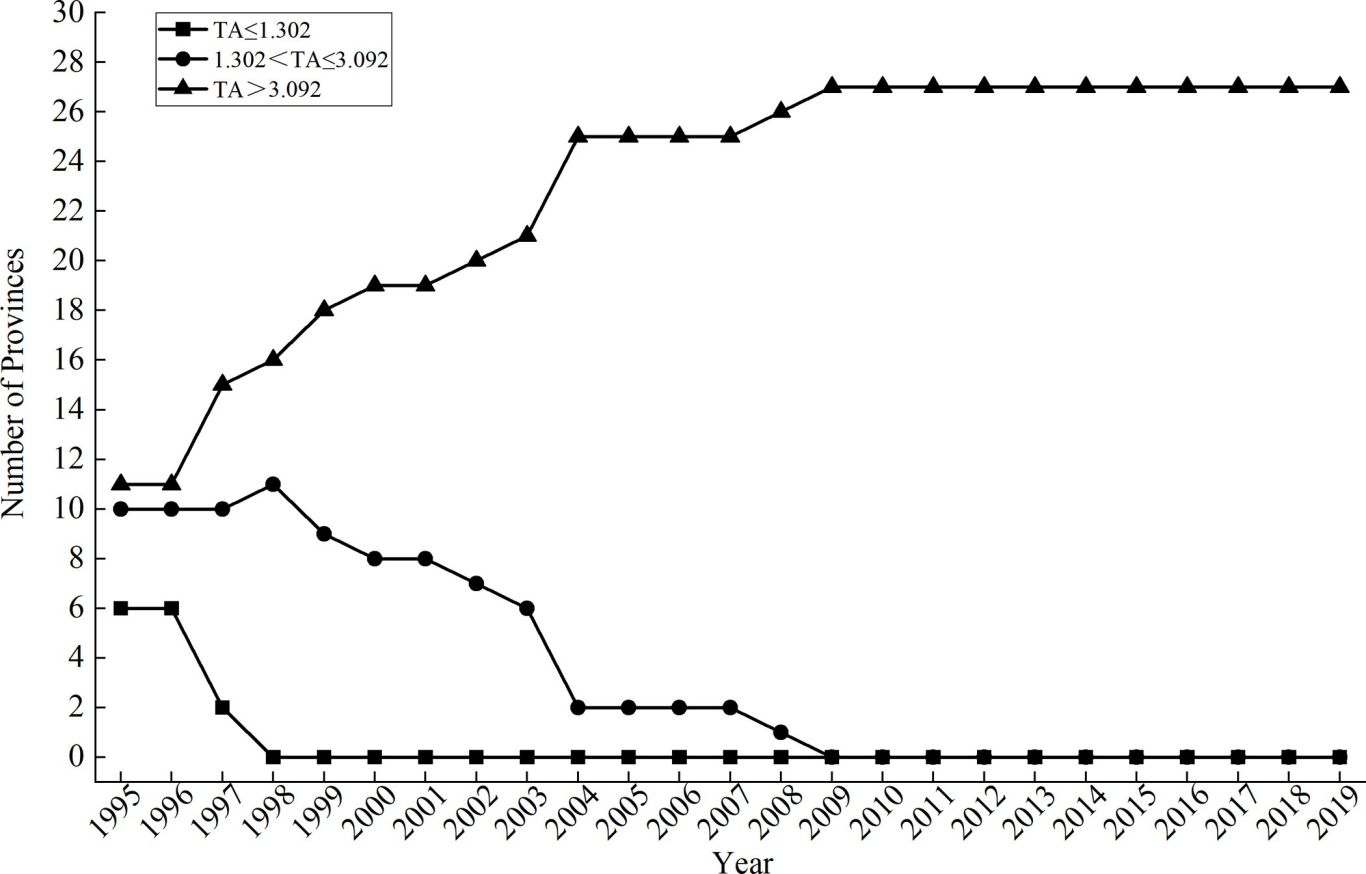
Time trend of the number of provinces in different technology accumulation intervals.

In order to more intuitively investigate the changes in the level of technology accumulation in China’s pharmaceutical manufacturing industry from 1995 to 2019, the trend chart of the number of provinces in different technology accumulation intervals is drawn, as shown in Fig 3.

It can be seen from Fig 3 that from 1995 to 1997, most provinces of China’s pharmaceutical manufacturing industry concentrated in the mid-tech accumulation (1.302<TA≤3.092) and high-tech accumulation (TA>3.092) intervals. Since 1998, there have been no provinces in the low-tech accumulation zone, which provides a good foundation for independent R&D activities in the pharmaceutical manufacturing industry and effectively promotes innovation performance. After 2009, the pharmaceutical manufacturing industry in all provinces is in the range of high-tech accumulation (TA>3.092). At this time, independent research and development have a reduced role in promoting innovation performance. These changes require sufficient attention.

### Robustness check

This paper uses the research method of Huang and Dong [37] to test the robustness of the estimation results by gradually adding control variables. The test results are shown in the S1 Appendix. It can be seen that the estimated results of adding the control variables RDP, TI, TS, and TR (models 1 to 4) in turn show that the technology accumulation threshold and confidence interval between independent R&D and innovation performance have not changed. The influence coefficients (${\hat{\alpha }}_{1}$, ${\hat{\alpha }}_{2}$, ${\hat{\alpha }}_{3}$) of independent R&D in the three technological accumulation intervals on innovation performance have not changed much. Although the values fluctuate slightly, the signs and significance have not changed, consistent with the previous estimation results. The influence coefficient of each control variable on innovation performance fluctuates slightly, but the sign and significance level are consistent with the previous estimation results. The above analysis results show that the previous estimation results are robust.

## Results and discussion

The contribution of independent R&D to innovation performance is limited by the level of technology accumulation, which tends to increase and then decrease as technology accumulation crosses the corresponding threshold value, consistent with the findings of Guo and Sun (2020) [17]. This could be explained by the fact that the accumulation of industrial technology is the knowledge resource of the enterprise, and it is also the necessary condition and internal foundation for independent research and development activities. When technology accumulation is low, it is not conducive to developing innovation activities, making independent R&D investment less effective in promoting performance. The continuous improvement of technology accumulation can improve the efficiency of enterprises’ technology search and selection in related fields and then effectively identify market opportunities so that independent R&D investment has increased the promotion of innovation performance. When technology accumulation is too high, it is easy to form technological path dependence and organizational rigidity [38]. Gilbert believes that the organization’s existing technology and knowledge promote the organization to strengthen self-learning. However, the accumulation of too many similar technologies and knowledge will not be conducive to new technologies and knowledge and will reduce the marginal benefits of independent R&D input and output [39].

At present, the technology accumulation level of China’s pharmaceutical manufacturing industry is in the high range, and the driving effect of its independent R&D on innovation performance shows a marginal diminishing state. On the one hand, it may be because the level of technology accumulation is too high, which is prone to path dependence. The accumulation of too many similar technologies hinders new technologies, reducing the marginal income of independent R&D input and output. On the other hand, it may be because studies have shown that the number of patent applications and authorizations in China’s pharmaceutical industry in recent years has reached the top of the world, but there are few essential and original high-quality patents, and generic drugs are still the mainstay [40]. Accumulating too much low-quality knowledge and technology will also affect independent research and development activities, resulting in a low conversion rate that cannot effectively improve innovation performance.

## Conclusion

The study aims to examine the relationship between independent R&D, technology accumulation, and innovation performance of China’s pharmaceutical manufacturing industry over the period of 1995~2019. The main conclusions are summarized as follows:

1. The contribution of independent R&D to innovation performance is limited by the level of technology accumulation, and there is a double-threshold effect based on the level of technology accumulation. With the level of technology accumulation continuously crossing the corresponding threshold, it shows a trend of rising and then falling.
2. At present, the level of technology accumulation in China’s pharmaceutical manufacturing industry is in the high technology accumulation range, and the contribution of independent R&D to innovation performance is in a marginal decreasing state.
3. R&D personnel investment and technology purchase in China’s pharmaceutical manufacturing industry can stimulate innovation performance, while technology introduction and technology adaptation has no significant effect on innovation performance.

This study offers several possible theoretical as well as practical implications. The theoretical contribution of this study is as follows:

First, this study incorporates independent R&D, technology accumulation, and innovation performance into a unified framework. Second, this study can be viewed as extending the current literature by examining the differences in the effects of independent R&D on innovation performance under different levels of technology accumulation.

Based on the findings of this study, some practical implications are put forward as follows:

1. Pharmaceutical companies need to maintain their technology accumulation at a reasonable level from a strategic point of view to facilitate independent R&D-driven innovation performance. When the level of technology accumulation is low, R&D activities can be carried out by seeking external cooperation to accumulate more knowledge and technology to lay a good foundation for independent R&D activities. When the level of technology accumulation is too high, it is important to be wary of forming technology path dependence and focus on quality innovation.
2. Pharmaceutical companies should establish the concept of innovation and increase investment in R&D to promote innovation performance, while policymakers should make innovation-induced policies to promote pharmaceutical companies R&D investment.
3. Pharmaceutical companies should increase investment in R&D personnel, purchase more advanced technologies and transform them into new products to meet market demand to enhance innovation performance further. In addition, pharmaceutical companies need to improve their ability to digest, absorb and transform advanced foreign technologies.

## Limitations of this study

This study has some limitations. First, independent R&D-driven innovation performance is a complex systematic process that may also be influenced by other factors beyond the control variables selected for this study. More factors can be incorporated into future studies with the continuous improvement of data sources and measurement methods. Secondly, we focus only on China’s pharmaceutical manufacturing industry, and other countries should also be included to compare with the findings of this study, and the scope of the study should be further broadened in the future.

## Supporting information

S1 Appendix (DOCX)

S2 Appendix (DOCX)
