# Supplementary material for: Independent research and development, technology accumulation and innovation performance: Evidence from China’s pharmaceutical manufacturing industry
Source: PLoS One. 2022 Apr 7;17(4):e0266768. doi: 10.1371/journal.pone.0266768 (PMC8989362; doi:10.1371/journal.pone.0266768)
Supplement: S1 Appendix — (DOCX) [file pone.0266768.s001.docx]

**Appendix. Robustness Check Results**

| **model** | **1** | **2** | **3** | **4** |
| --- | --- | --- | --- | --- |
| **A：Threshold estimation** | | | | |
| ${\hat{\boldsymbol{\gamma}}}_{\boldsymbol{1}}$ **95% confidence interval** | 1.302  [1.216 , 1.332] | 1.302  [1.216 , 1.332] | 1.302  [1.216 , 1.332] | 1.302  [1.216 , 1.332] |
| ${\hat{\boldsymbol{\gamma}}}_{\boldsymbol{2}}$ **95% confidence interval** | 3.092  [3.044 , 3.133] | 3.092  [3.044 , 3.133] | 3.092  [3.044 , 3.133] | 3.092  [3.044 , 3.133] |
| **B：The impact of independent R&D on innovation performance** | | | | |
| ${\hat{\boldsymbol{\alpha}}}_{\boldsymbol{1}}$ | 0.261^**^ | 0.310^**^ | 0.280^**^ | 0.235^**^ |
| ${\hat{\boldsymbol{\alpha}}}_{\boldsymbol{2}}$ | 0.550^***^ | 0.514^***^ | 0.446^***^ | 0.474^***^ |
| ${\hat{\boldsymbol{\alpha}}}_{\boldsymbol{3}}$ | 0.369^***^ | 0.351^***^ | 0.301^***^ | 0.315^***^ |
| **C：The influence of control variables on innovation performance** | | | | |
| **L1.Q** | 0.243^***^ | 0.252^***^ | 0.268^***^ | 0.300^***^ |
| **RDP** | 0.428^***^ | 0.426^***^ | 0.399^***^ | 0.367^***^ |
| **TI** |  | 0.023 | 0.002 | 0.021 |
| **TS** |  |  | 0.068^**^ | 0.073^**^ |
| **TR** |  |  |  | -0.190 |

**Notes: ** p < 0.05, ***p < 0.01**
