# Supplementary material for: Independent research and development, technology accumulation and innovation performance: Evidence from China’s pharmaceutical manufacturing industry
Source: PLoS One. 2022 Apr 7;17(4):e0266768. doi: 10.1371/journal.pone.0266768 (PMC8989362; doi:10.1371/journal.pone.0266768)
Supplement: S2 Appendix — (DOCX) [file pone.0266768.s002.docx]

**Introduction to research and development (R&D)**

R&D refers to creative, systematic work to increase the stock of knowledge (also including knowledge about humans, culture, and society) and to design new applications of existing knowledge, and includes three types of basic research, applied research, and experimental development. Basic and applied research are collectively referred to as scientific research. R&D activities should satisfy five conditions: novelty, creativity, uncertainty, systematicity, and transferability (replicability).

Basic research is an experimental or theoretical work that does not presuppose any specific purpose of the application or use, and its primary purpose is to obtain basic principles, laws, and new knowledge of (already occurring) phenomena and observable facts. The results of basic research are usually expressed as the formulation of general principles, theories, or laws and are mainly in papers, publications, research reports, etc. Basic research includes pure basic research and directed basic research.

Pure basic research is basic research that does not pursue economic or social benefits, nor does it seek to apply the results, but only to add new knowledge. Targeted basic research is basic research that provides basic knowledge in some aspects for the identification and solution of currently known or future predictable problems.

Applied research is initial research conducted to acquire new knowledge to achieve a specific practical purpose or goal. Applied research is conducted to determine possible uses of basic research results or identify new methods for achieving a specific and predetermined goal. Its research results are mainly in papers, publications, research reports, schematic models, or patents for inventions.

Experimental development is systematic research conducted to develop new products and processes or improve existing products and processes using knowledge gained from scientific research, practical experience, and other knowledge generated in the research process. The research results are mainly in patents, proprietary technologies, product prototypes, original prototypes, and devices with novelty.
